# 1-Benzyl-3-(2-methoxy­phen­yl)imidazolium tetra­fluoro­borate

**DOI:** 10.1107/S1600536809031602

**Published:** 2009-08-19

**Authors:** Ping Jiang

**Affiliations:** a School of Chemistry and Chemical Engineering, China West Normal University, Nanchong 637002, People’s Republic of China

## Abstract

In the title compound, C_17_H_17_N_2_O^+^·BF_4_^−^, the central imidazolium ring makes dihedral angles of 74.58 (9) and 40.10 (6)° with the phenyl and 2-methoxy­phenyl rings, respectively. In the crystal, a strong π–π inter­action is observed between the imidazolium and 2-methoxy­phenyl rings, with a centroid–centroid distance of 3.5115 (15) Å. In addition, C—H⋯F and C—H⋯O hydrogen bonds and C—H⋯π inter­actions involving the ­phenyl ring are observed.

## Related literature

For the synthesis, see: Liu *et al.*. (2003 ▶). For general background to *N*-heterocyclic carbenes, see: Arduengo *et al.* (1991 ▶). For the biological activity of imidazolium salts, see: Vik *et al.* (2007 ▶); Demberelnyamba *et al.* (2004 ▶); Dallas *et al.* (2007 ▶); Ballistreri *et al.* (2004 ▶).
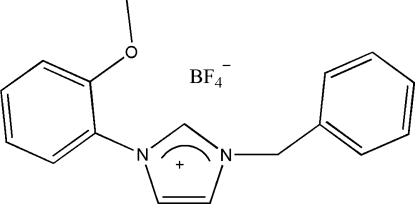


## Experimental

### 

#### Crystal data

C_17_H_17_N_2_O^+^·BF_4_^−^
*M* *_r_* = 352.14
Orthorhombic, 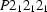

*a* = 7.1880 (13) Å
*b* = 15.114 (3) Å
*c* = 15.358 (3) Å
*V* = 1668.4 (5) Å^3^
*Z* = 4
Mo *K*α radiation
μ = 0.12 mm^−1^
*T* = 93 K
0.40 × 0.33 × 0.33 mm

#### Data collection

Rigaku SPIDER diffractometer
Absorption correction: none
13633 measured reflections
2197 independent reflections
2069 reflections with *I* > 2σ(*I*)
*R*_int_ = 0.032

#### Refinement

*R*[*F* ^2^ > 2σ(*F* ^2^)] = 0.044
*wR*(*F* ^2^) = 0.116
*S* = 1.00
2197 reflections
227 parameters
H-atom parameters constrained
Δρ_max_ = 0.85 e Å^−3^
Δρ_min_ = −0.19 e Å^−3^

### 

Data collection: *RAPID-AUTO* (Rigaku/MSC, 2004 ▶); cell refinement: *RAPID-AUTO*; data reduction: *RAPID-AUTO*; program(s) used to solve structure: *SHELXS97* (Sheldrick, 2008 ▶); program(s) used to refine structure: *SHELXL97* (Sheldrick, 2008 ▶); molecular graphics: *SHELXTL* (Sheldrick, 2008 ▶); software used to prepare material for publication: *SHELXL97*.

## Supplementary Material

Crystal structure: contains datablocks global, I. DOI: 10.1107/S1600536809031602/ci2870sup1.cif

Structure factors: contains datablocks I. DOI: 10.1107/S1600536809031602/ci2870Isup2.hkl

Additional supplementary materials:  crystallographic information; 3D view; checkCIF report

## Figures and Tables

**Table 1:** Hydrogen-bond geometry (Å, °)

*D*—H⋯*A*	*D*—H	H⋯*A*	*D*⋯*A*	*D*—H⋯*A*
C7—H7*B*⋯F3^i^	0.99	2.30	3.268 (3)	167
C8—H8⋯F4^i^	0.95	2.34	3.220 (3)	154
C9—H9⋯F1	0.95	2.21	3.122 (3)	161
C10—H10⋯F1^ii^	0.95	2.37	3.236 (3)	150
C10—H10⋯F2^ii^	0.95	2.38	3.221 (3)	147
C12—H12⋯O1^ii^	0.95	2.57	3.292 (3)	133
C14—H14⋯F3^iii^	0.95	2.48	3.274 (3)	140
C13—H13⋯*Cg*1^ii^	0.94	2.61	3.460 (3)	149

Symmetry codes: (i) 
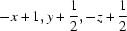
; (ii) 
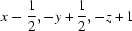
; (iii) 
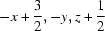
. *Cg*1 is the centroid of the C1–C6 ring.

**Fig. 1.**
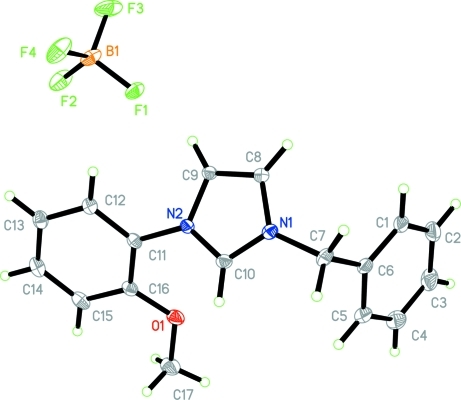
The molecular structure of the title compound, showing 30% probability displacement ellipsoids and the atomic numbering scheme.

## supplementary crystallographic information

### Comment

Since the synthesis and isolation of the first stable, crystalline N-heterocyclic carbene (NHC) was disclosed by Arduengo *et al.* (1991) scientists have paid much attention to this field. In recent years, a large number of N-heterocyclic carbene (NHC) precursors have been synthesized 1,3-Disubstituted imidazolium salts are potential precursors for the synthesis of various transition metal NHCs. In addition, a number of biological activities of imidazolium salts have been reported including antimicrobial, antifungal, antitumor activities (Vik *et al.*, 2007; Demberelnyamba *et al.*, 2004; Dallas *et al.*, 2007; Ballistreri *et al.*, 2004). We report here crystal structure of a NHC precursor, the title compound.

Bond lengths and angles in title molecule (Fig. 1) are normal. The imidazolium ring makes dihedral angles of 74.58 (9)° and 40.10 (6)°, respectively, with the phenyl ring and 2-methoxyphenyl ring. The methoxy group is slightly twisted away from the attached ring [C17—O1—C16—C15 = -7.8 (4)°].

In the crystal, there are strong π-π interactions between imidazolium and 2-methoxyphenyl rings, with a *Cg*2···*Cg*2^ii^ distance of 3.5115 (15) Å [symmetry code: (ii) *x* - 1/2, 1/2 - *y*, 1 - *z*] where *Cg*2 and *Cg*3 are centroids of the imidazolium and methoxyphenyl rings, respectively. In addition, C—H···F and C—H···O hydrogen bonds and C—H···π interactions involving the C1—C6 phenyl ring are observed (Table 1).

### Experimental

The title compound was prepared according to the reported procedure of Liu *et al.*. (2003). Colourless single crystals suitable for X-ray diffraction were obtained by recrystallization from dichloromethane and petroleum ether.

### Refinement

H atoms were placed in calculated positions [C—H = 0.95–0.99 Å] and refined using a riding model, with *U*_iso_(H) = 1.2*U*_eq_(C).

### Crystal data

**Table:**

C_17_H_17_N_2_O^+^·BF_4_^−^	*F*(000) = 728
*M**_r_* = 352.14	*D*_x_ = 1.402 Mg m^−^^3^
Orthorhombic, *P*2_1_2_1_2_1_	Mo *K*α radiation, λ = 0.71073 Å
Hall symbol: P 2ac 2ab	Cell parameters from 5660 reflections
*a* = 7.1880 (13) Å	θ = 3.0–27.5°
*b* = 15.114 (3) Å	µ = 0.12 mm^−^^1^
*c* = 15.358 (3) Å	*T* = 93 K
*V* = 1668.4 (5) Å^3^	Block, colourless
*Z* = 4	0.40 × 0.33 × 0.33 mm

### Data collection

**Table:**

Rigaku SPIDER diffractometer	2069 reflections with *I* > 2σ(*I*)
Radiation source: Rotating Anode	*R*_int_ = 0.032
graphite	θ_max_ = 27.5°, θ_min_ = 3.0°
ω scans	*h* = −9→9
13633 measured reflections	*k* = −19→19
2197 independent reflections	*l* = −19→15

### Refinement

**Table:**

Refinement on *F*^2^	Primary atom site location: structure-invariant direct methods
Least-squares matrix: full	Secondary atom site location: difference Fourier map
*R*[*F*^2^ > 2σ(*F*^2^)] = 0.044	Hydrogen site location: inferred from neighbouring sites
*wR*(*F*^2^) = 0.116	H-atom parameters constrained
*S* = 1.00	*w* = 1/[σ^2^(*F*_o_^2^) + (0.068*P*)^2^ + 0.46*P*] where *P* = (*F*_o_^2^ + 2*F*_c_^2^)/3
2197 reflections	(Δ/σ)_max_ = 0.001
227 parameters	Δρ_max_ = 0.85 e Å^−^^3^
0 restraints	Δρ_min_ = −0.18 e Å^−^^3^

### Special details

**Table:**

Geometry. All e.s.d.'s (except the e.s.d. in the dihedral angle between two l.s. planes) are estimated using the full covariance matrix. The cell e.s.d.'s are taken into account individually in the estimation of e.s.d.'s in distances, angles and torsion angles; correlations between e.s.d.'s in cell parameters are only used when they are defined by crystal symmetry. An approximate (isotropic) treatment of cell e.s.d.'s is used for estimating e.s.d.'s involving l.s. planes.
Refinement. Refinement of *F*^2^ against ALL reflections. The weighted *R*-factor *wR* and goodness of fit *S* are based on *F*^2^, conventional *R*-factors *R* are based on *F*, with *F* set to zero for negative *F*^2^. The threshold expression of *F*^2^ > σ(*F*^2^) is used only for calculating *R*-factors(gt) *etc*. and is not relevant to the choice of reflections for refinement. *R*-factors based on *F*^2^ are statistically about twice as large as those based on *F*, and *R*- factors based on ALL data will be even larger.

### Fractional atomic coordinates and isotropic or equivalent isotropic displacement parameters (Å^2^)

**Table:**

	*x*	*y*	*z*	*U*_iso_*/*U*_eq_	
B1	0.6858 (5)	0.1082 (2)	0.2432 (2)	0.0349 (7)	
F1	0.7005 (2)	0.18357 (10)	0.29508 (10)	0.0360 (4)	
F2	0.7661 (3)	0.03715 (11)	0.28690 (12)	0.0520 (5)	
F3	0.7771 (3)	0.12215 (13)	0.16519 (11)	0.0601 (6)	
F4	0.4996 (3)	0.09039 (13)	0.22721 (13)	0.0555 (6)	
O1	0.6091 (3)	0.27395 (12)	0.65713 (11)	0.0314 (4)	
N1	0.4482 (3)	0.45145 (13)	0.48559 (13)	0.0226 (4)	
N2	0.4930 (3)	0.31030 (12)	0.49196 (12)	0.0203 (4)	
C1	0.6888 (4)	0.63622 (15)	0.48138 (17)	0.0291 (5)	
H1	0.6486	0.6402	0.4226	0.035*	
C2	0.8534 (5)	0.67656 (17)	0.5060 (2)	0.0390 (7)	
H2	0.9262	0.7075	0.4642	0.047*	
C3	0.9113 (5)	0.67162 (19)	0.5918 (2)	0.0453 (8)	
H3	1.0237	0.6996	0.6091	0.054*	
C4	0.8069 (5)	0.6263 (2)	0.6522 (2)	0.0460 (8)	
H4	0.8471	0.6232	0.7111	0.055*	
C5	0.6423 (5)	0.58515 (17)	0.62717 (18)	0.0359 (6)	
H5	0.5710	0.5535	0.6689	0.043*	
C6	0.5814 (4)	0.59003 (16)	0.54127 (16)	0.0264 (5)	
C7	0.4059 (4)	0.54316 (16)	0.51345 (18)	0.0289 (5)	
H7A	0.3167	0.5419	0.5626	0.035*	
H7B	0.3475	0.5758	0.4647	0.035*	
C8	0.5203 (3)	0.42622 (15)	0.40606 (16)	0.0230 (5)	
H8	0.5458	0.4638	0.3579	0.028*	
C9	0.5478 (3)	0.33788 (15)	0.40976 (15)	0.0211 (5)	
H9	0.5956	0.3015	0.3646	0.025*	
C10	0.4336 (3)	0.38076 (16)	0.53617 (15)	0.0227 (5)	
H10	0.3883	0.3803	0.5943	0.027*	
C11	0.4976 (3)	0.22050 (15)	0.52293 (15)	0.0213 (5)	
C12	0.4486 (3)	0.15283 (16)	0.46744 (16)	0.0235 (5)	
H12	0.4116	0.1657	0.4094	0.028*	
C13	0.4533 (3)	0.06574 (16)	0.49642 (18)	0.0288 (5)	
H13	0.4208	0.0188	0.4582	0.035*	
C14	0.5056 (4)	0.04793 (17)	0.58102 (19)	0.0317 (6)	
H14	0.5070	−0.0116	0.6010	0.038*	
C15	0.5562 (3)	0.11531 (17)	0.63726 (18)	0.0305 (6)	
H15	0.5918	0.1019	0.6953	0.037*	
C16	0.5549 (3)	0.20287 (16)	0.60874 (15)	0.0246 (5)	
C17	0.6499 (5)	0.2592 (2)	0.74763 (17)	0.0423 (7)	
H17A	0.5385	0.2369	0.7771	0.051*	
H17B	0.6885	0.3151	0.7746	0.051*	
H17C	0.7504	0.2158	0.7529	0.051*	

### Atomic displacement parameters (Å^2^)

**Table:**

	*U*^11^	*U*^22^	*U*^33^	*U*^12^	*U*^13^	*U*^23^
B1	0.0408 (16)	0.0351 (15)	0.0287 (15)	0.0150 (14)	−0.0110 (14)	−0.0110 (13)
F1	0.0441 (9)	0.0332 (8)	0.0308 (8)	0.0046 (7)	0.0012 (7)	−0.0122 (7)
F2	0.0711 (13)	0.0394 (9)	0.0455 (11)	0.0217 (9)	−0.0260 (10)	−0.0105 (8)
F3	0.0828 (15)	0.0669 (13)	0.0306 (10)	0.0258 (12)	0.0078 (10)	−0.0132 (9)
F4	0.0473 (10)	0.0555 (11)	0.0638 (13)	0.0119 (10)	−0.0236 (10)	−0.0256 (10)
O1	0.0325 (10)	0.0403 (10)	0.0214 (9)	−0.0103 (8)	−0.0031 (8)	0.0036 (7)
N1	0.0221 (9)	0.0230 (9)	0.0228 (10)	0.0009 (8)	0.0009 (8)	−0.0030 (8)
N2	0.0206 (9)	0.0208 (9)	0.0194 (9)	−0.0025 (7)	0.0009 (8)	0.0011 (7)
C1	0.0393 (13)	0.0219 (10)	0.0261 (13)	0.0020 (11)	−0.0057 (11)	0.0006 (10)
C2	0.0464 (16)	0.0290 (13)	0.0415 (16)	−0.0099 (12)	−0.0037 (14)	0.0027 (11)
C3	0.0506 (18)	0.0347 (14)	0.0504 (18)	−0.0131 (13)	−0.0161 (15)	−0.0031 (13)
C4	0.065 (2)	0.0432 (16)	0.0298 (15)	−0.0067 (16)	−0.0153 (15)	−0.0046 (13)
C5	0.0518 (17)	0.0313 (12)	0.0245 (13)	−0.0014 (13)	0.0000 (13)	−0.0046 (10)
C6	0.0333 (13)	0.0212 (10)	0.0247 (12)	0.0035 (10)	−0.0001 (10)	−0.0039 (9)
C7	0.0308 (12)	0.0237 (11)	0.0321 (14)	0.0052 (10)	0.0025 (11)	−0.0077 (10)
C8	0.0252 (11)	0.0238 (10)	0.0200 (11)	−0.0005 (9)	0.0021 (10)	−0.0004 (9)
C9	0.0220 (11)	0.0225 (10)	0.0187 (11)	0.0002 (9)	0.0019 (9)	0.0000 (9)
C10	0.0214 (10)	0.0265 (11)	0.0202 (11)	−0.0009 (9)	0.0030 (9)	−0.0009 (9)
C11	0.0150 (9)	0.0244 (10)	0.0245 (12)	−0.0011 (9)	0.0010 (9)	0.0044 (9)
C12	0.0181 (10)	0.0250 (11)	0.0275 (13)	0.0007 (9)	−0.0002 (9)	0.0017 (9)
C13	0.0233 (11)	0.0254 (11)	0.0377 (15)	−0.0009 (10)	0.0034 (11)	0.0012 (10)
C14	0.0242 (11)	0.0274 (11)	0.0435 (15)	0.0009 (10)	0.0027 (12)	0.0092 (11)
C15	0.0218 (11)	0.0378 (13)	0.0321 (14)	0.0019 (10)	−0.0002 (10)	0.0134 (11)
C16	0.0179 (10)	0.0328 (12)	0.0230 (12)	−0.0045 (10)	−0.0004 (9)	0.0038 (9)
C17	0.0478 (17)	0.0537 (17)	0.0253 (14)	−0.0128 (15)	−0.0092 (13)	0.0070 (13)

### Geometric parameters (Å, °)

**Table:**

B1—F3	1.382 (4)	C5—H5	0.95
B1—F4	1.388 (4)	C6—C7	1.509 (4)
B1—F2	1.392 (3)	C7—H7A	0.99
B1—F1	1.394 (3)	C7—H7B	0.99
O1—C16	1.363 (3)	C8—C9	1.351 (3)
O1—C17	1.438 (3)	C8—H8	0.95
N1—C10	1.325 (3)	C9—H9	0.95
N1—C8	1.381 (3)	C10—H10	0.95
N1—C7	1.482 (3)	C11—C12	1.377 (3)
N2—C10	1.333 (3)	C11—C16	1.406 (3)
N2—C9	1.387 (3)	C12—C13	1.390 (3)
N2—C11	1.439 (3)	C12—H12	0.95
C1—C2	1.383 (4)	C13—C14	1.379 (4)
C1—C6	1.389 (4)	C13—H13	0.95
C1—H1	0.95	C14—C15	1.384 (4)
C2—C3	1.385 (4)	C14—H14	0.95
C2—H2	0.95	C15—C16	1.394 (3)
C3—C4	1.376 (5)	C15—H15	0.95
C3—H3	0.95	C17—H17A	0.98
C4—C5	1.391 (5)	C17—H17B	0.98
C4—H4	0.95	C17—H17C	0.98
C5—C6	1.392 (4)		
			
F3—B1—F4	109.5 (2)	C6—C7—H7B	109.6
F3—B1—F2	109.8 (3)	H7A—C7—H7B	108.1
F4—B1—F2	109.6 (3)	C9—C8—N1	106.9 (2)
F3—B1—F1	109.6 (3)	C9—C8—H8	126.5
F4—B1—F1	109.4 (2)	N1—C8—H8	126.5
F2—B1—F1	108.9 (2)	C8—C9—N2	107.1 (2)
C16—O1—C17	117.6 (2)	C8—C9—H9	126.5
C10—N1—C8	109.00 (19)	N2—C9—H9	126.5
C10—N1—C7	124.7 (2)	N1—C10—N2	108.67 (19)
C8—N1—C7	126.2 (2)	N1—C10—H10	125.7
C10—N2—C9	108.33 (19)	N2—C10—H10	125.7
C10—N2—C11	126.33 (19)	C12—C11—C16	121.0 (2)
C9—N2—C11	125.33 (19)	C12—C11—N2	119.4 (2)
C2—C1—C6	121.1 (2)	C16—C11—N2	119.7 (2)
C2—C1—H1	119.5	C11—C12—C13	119.9 (2)
C6—C1—H1	119.5	C11—C12—H12	120.0
C1—C2—C3	119.6 (3)	C13—C12—H12	120.0
C1—C2—H2	120.2	C14—C13—C12	119.6 (2)
C3—C2—H2	120.2	C14—C13—H13	120.2
C4—C3—C2	120.3 (3)	C12—C13—H13	120.2
C4—C3—H3	119.8	C13—C14—C15	121.1 (2)
C2—C3—H3	119.8	C13—C14—H14	119.5
C3—C4—C5	120.0 (3)	C15—C14—H14	119.5
C3—C4—H4	120.0	C14—C15—C16	120.1 (2)
C5—C4—H4	120.0	C14—C15—H15	120.0
C4—C5—C6	120.4 (3)	C16—C15—H15	120.0
C4—C5—H5	119.8	O1—C16—C15	125.1 (2)
C6—C5—H5	119.8	O1—C16—C11	116.4 (2)
C1—C6—C5	118.7 (3)	C15—C16—C11	118.4 (2)
C1—C6—C7	120.9 (2)	O1—C17—H17A	109.5
C5—C6—C7	120.4 (2)	O1—C17—H17B	109.5
N1—C7—C6	110.4 (2)	H17A—C17—H17B	109.5
N1—C7—H7A	109.6	O1—C17—H17C	109.5
C6—C7—H7A	109.6	H17A—C17—H17C	109.5
N1—C7—H7B	109.6	H17B—C17—H17C	109.5
			
C6—C1—C2—C3	−0.6 (4)	C9—N2—C10—N1	0.1 (3)
C1—C2—C3—C4	0.5 (5)	C11—N2—C10—N1	−179.0 (2)
C2—C3—C4—C5	0.1 (5)	C10—N2—C11—C12	139.7 (2)
C3—C4—C5—C6	−0.5 (5)	C9—N2—C11—C12	−39.2 (3)
C2—C1—C6—C5	0.2 (4)	C10—N2—C11—C16	−41.3 (3)
C2—C1—C6—C7	−177.6 (2)	C9—N2—C11—C16	139.7 (2)
C4—C5—C6—C1	0.4 (4)	C16—C11—C12—C13	0.8 (3)
C4—C5—C6—C7	178.2 (3)	N2—C11—C12—C13	179.7 (2)
C10—N1—C7—C6	96.0 (3)	C11—C12—C13—C14	0.6 (4)
C8—N1—C7—C6	−79.3 (3)	C12—C13—C14—C15	−1.0 (4)
C1—C6—C7—N1	89.0 (3)	C13—C14—C15—C16	0.0 (4)
C5—C6—C7—N1	−88.8 (3)	C17—O1—C16—C15	−7.8 (4)
C10—N1—C8—C9	0.4 (3)	C17—O1—C16—C11	173.7 (2)
C7—N1—C8—C9	176.3 (2)	C14—C15—C16—O1	−177.2 (2)
N1—C8—C9—N2	−0.4 (3)	C14—C15—C16—C11	1.4 (4)
C10—N2—C9—C8	0.2 (3)	C12—C11—C16—O1	176.9 (2)
C11—N2—C9—C8	179.3 (2)	N2—C11—C16—O1	−2.0 (3)
C8—N1—C10—N2	−0.3 (3)	C12—C11—C16—C15	−1.7 (3)
C7—N1—C10—N2	−176.3 (2)	N2—C11—C16—C15	179.4 (2)

### Hydrogen-bond geometry (Å, °)

**Table:**

*D*—H···*A*	*D*—H	H···*A*	*D*···*A*	*D*—H···*A*
C7—H7B···F3^i^	0.99	2.30	3.268 (3)	167
C8—H8···F4^i^	0.95	2.34	3.220 (3)	154
C9—H9···F1	0.95	2.21	3.122 (3)	161
C10—H10···F1^ii^	0.95	2.37	3.236 (3)	150
C10—H10···F2^ii^	0.95	2.38	3.221 (3)	147
C12—H12···O1^ii^	0.95	2.57	3.292 (3)	133
C14—H14···F3^iii^	0.95	2.48	3.274 (3)	140
C13—H13···Cg1^ii^	0.94	2.61	3.460 (3)	149

Symmetry codes: (i) −*x*+1, *y*+1/2, −*z*+1/2;  (ii) *x*−1/2, −*y*+1/2, −*z*+1; (iii) −*x*+3/2, −*y*, *z*+1/2.
